# Heme Burden and Ensuing Mechanisms That Protect the Kidney: Insights from Bench and Bedside

**DOI:** 10.3390/ijms22158174

**Published:** 2021-07-30

**Authors:** József Balla, Abolfazl Zarjou

**Affiliations:** 1 ELKH-UD Vascular Biology and Myocardial Pathophysiology Research Group, Division of Nephrology, Department of Medicine, Faculty of Medicine, Hungarian Academy of Sciences, H-4032 Debrecen, Hungary; balla@belklinika.com; 2 Nephrology Research and Training Center, Division of Nephrology, Department of Medicine, University of Alabama at Birmingham, 618 Zeigler Research Building, 703 South 19th Street, Birmingham, AL 35294, USA

**Keywords:** heme oxygenase-1, ferritin, rhabdomyolysis, hemolysis, kidney disease

## Abstract

With iron at its core, the tetrapyrrole heme ring is a cardinal prosthetic group made up of many proteins that participate in a wide array of cellular functions and metabolism. Once released, due to its pro-oxidant properties, free heme in sufficient amounts can result in injurious effects to the kidney and other organs. Heme oxygenase-1 (HO-1) has evolved to promptly attend to such injurious potential by facilitating degradation of heme into equimolar amounts of carbon monoxide, iron, and biliverdin. HO-1 induction is a beneficial response to tissue injury in diverse animal models of diseases, including those that affect the kidney. These protective attributes are mainly due to: (i) prompt degradation of heme leading to restraining potential hazardous effects of free heme, and (ii) generation of byproducts that along with induction of ferritin have proven beneficial in a number of pathological conditions. This review will focus on describing clinical aspects of some of the conditions with the unifying end-result of increased heme burden and will discuss the molecular mechanisms that ensue to protect the kidneys.

## 1. Introduction

Heme is an evolutionarily conserved, ubiquitous iron-containing compound that is essential in numerous cellular functions [1,2,3]. However, heme is strongly hydrophobic and hence can readily enter cell membranes increasing cellular susceptibility to oxidant-mediated damage based on its reactivity and pro-oxidant properties [2,4]. There are hundreds of heme proteins, and heme is present in virtually all cellular compartments as a requisite for aerobic life [1]. The vast majority of heme in mammalians is confined within hemoglobin and myoglobin. Under homeostatic conditions cellular heme levels are stringently controlled, a process that involves a well-orchestrated balance between heme biosynthesis and catabolism. However, during pathological conditions and upon injury, heme can be released and when present in sufficient amount leads to commencement of an injury cycle that could eventually lead to cellular death and organ failure [2]. As it pertains to the kidney, several clinical conditions have been recognized that are associated with significant amount of free heme and subsequent kidney damage [5,6,7]. The kidney is frequently involved during clinical settings, with the common denominator of increased heme burden given its primary function of filtration. Moreover, the proximal tubules possess a high number of mitochondria that upon injury release their cytochrome heme content leading to higher levels of local heme and hence potentiating the cycle of injury. Although the list of such clinical settings is vast and includes some frequent diseases and syndromes, massive intravascular hemolysis and rhabdomyolysis are the main culprits that may result in diverse forms of kidney disease, most commonly acute kidney injury (AKI) [5]. In fact, both myoglobin and hemoglobin are filtered by the glomerulus into the urinary space where they are degraded, thus releasing heme pigment. As a defense against such toxicity cells promptly upregulate heme oxygenase-1 (HO-1) and ferritin expression [5,8]. HO catalyzes the regiospecific and rate limiting step of heme degradation to carbon monoxide (CO), ferrous iron and biliverdin [9]. Biliverdin is subsequently converted to bilirubin by the action of biliverdin reductase, while ferrous iron stimulates the induction of ferritin. In mammalian systems, two distinct enzymes make up the HO family; namely HO-1 and HO-2, and these enzymes are products of distinct genes [7]. Furthermore, while HO-1 is rapidly induced in response to a number of stimuli, HO-2 expression is constitutive. Discoveries in the early 1990s that described anti-oxidant and protective attributes of HO-1 [10] and ferritin [11] has led to a remarkable and vast field of investigations with many exciting and seminal breakthroughs. The cytoprotective effects of HO-1 induction are primarily due to exerting anti-inflammatory, anti-apoptotic, anti-oxidant, and anti-proliferative effects as well as modulation of fibrosis and neovascularization [3,4,5,12]. Within the limitation of the review, we will only discuss some of the most common conditions associated with increased heme burden from a clinical perspective and will follow that with brief description of the literature about current understanding of the molecular mechanisms that confer protection against heme mediated toxicity and organ damage.

## 2. Clinical Significance

### 2.1. Rhabdomyolysis

**Figure 1:**
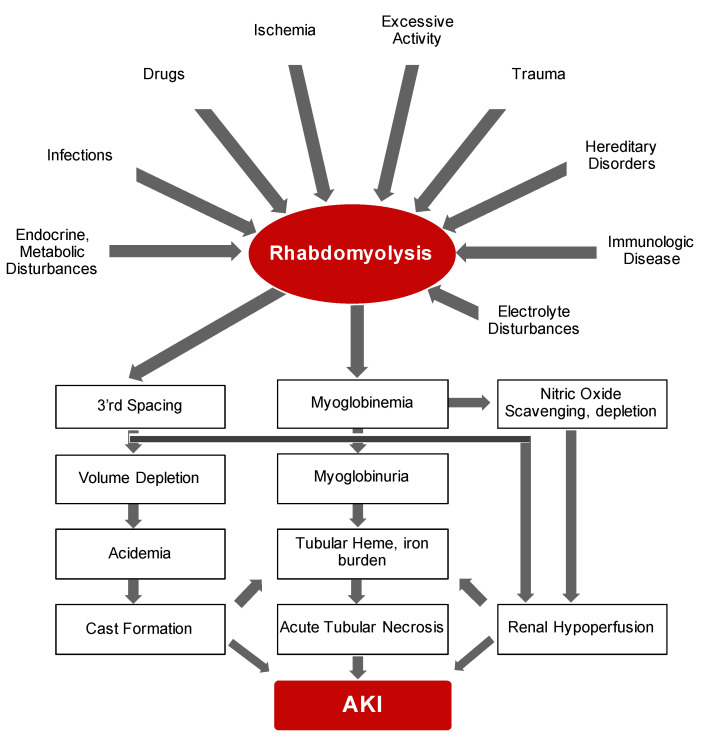
Simplified schematic demonstrates potential culprits and pathogenesis of rhabdomyolysis leading to AKI.

Bywaters and Beall documented the first description of the consequences of traumatic muscle injury on kidney function during air-raid casualties of World War II [13]. They documented four patients who suffered crush injuries with similar clinical features that included brown-black granular casts, oliguria, progressive rise in blood urea and potassium and eventual death. Bywaters and Stead were also able to reproduce these findings by injection of human myoglobin in rabbits on an “acidifying” diet with urinary pH of below 6.0 [14]. In simple terms, rhabdomyolysis is the necrosis of muscle and subsequent release of intracellular components into circulation. It is now evident that rhabdomyolysis and resultant myoglobinuric AKI is a common and potentially life-threatening syndrome characterized by release of toxic muscle cell contents into the circulation [15,16,17,18]. On the whole, the degree of muscle damage rather than initiating factor may determine the clinical course of rhabdomyolysis ranging from asymptomatic illness to a life-threatening condition. While the etiological range of rhabdomyolysis is broad and pathological sequelae is complex (summarized in Figure 1), the presence of multiple concomitant etiologic factors is not uncommon [19].

The major instigating factor of AKI is the release of myoglobin. The half-life of myoglobin is rather short mainly because it is not significantly protein-bound and hence is rapidly cleared by the kidneys and generally vanishes within six hours of release [20]. Therefore, the preferred diagnostic method of rhabdomyolysis is detection of creatine kinase, an intracellular enzyme, which has a serum half-life of around 36 h [21]. In addition to AKI, fluid and electrolyte abnormalities may also be noted during early presentation. With significant degree of muscle injury, extracellular fluid may shift into the damaged cells resulting in potential hypovolemia and hence further susceptibility to AKI. Release of high levels of intracellular phosphorus and potassium elevates their levels in circulation while dystrophic calcification may also lead to hypocalcemia. In addition, significant release of purines from damaged muscle cells and reduced kidney function, leading to diminished excretion, may also result in hyperuricemia. Disseminated intravascular coagulation, albeit rare, may be another consequence of severe rhabdomyolysis that is suggested to result from release pro-thrombotic molecules from damaged cells [22]. Taken together, rhabdomyolysis is a frequent, and—from an etiologic standpoint—a multifaceted clinical syndrome that requires prompt recognition and diagnosis and management of fluid and electrolyte abnormalities.

### 2.2. Hemolytic Anemias

Shortened survival of red blood cells (RBCs) may be the result of either inherent abnormalities in RBCs (intracorpuscular), which frequently result from genetic defects, or external causes (extracorpuscular) that are generally acquired (partial list of causes of hemolytic anemias is summarized in Table 1).

Irrespective of the etiology, intravascular hemolysis primes a sequence of events with the adverse outcomes that frequently involves systemic inflammation, vasomotor dysfunction, increased coagulopathy, proliferative vasculopathy and kidney injury [23,24,25]. The complexity of etiologies, pathophysiology, presentation, clinical classification and treatment approaches obviates the multifaceted nature of hemolytic anemias and necessitates further discussion in more elaborate and specific reviews. Accordingly, within the limitation of this review, we will only briefly discuss paroxysmal nocturnal hemoglobinuria (PNH) and malaria. It is of note that clinical aspects of sickle cell nephropathy, which is one of the most common causes of hereditary hemolytic anemias in the world, and its adverse consequences that literally affect all aspects of kidney function are discussed elsewhere [26,27,28].

### 2.3. Paroxysmal Nocturnal Hemoglobinuria (PNH)

PNH is a rare, potentially life-threatening hematopoietic stem cell condition leading to a myriad of clinical manifestations [29,30,31,32]. Given the low incidence and mild, nonspecific initial presentation, accurate diagnosis and management is delayed in many cases. The vast majority of cases are due to reduction in or absence of glycosylphosphatidylinositol-anchor on the cell surface [29,32]. This anchor plays a crucial role in linking multiple proteins to the plasma membrane of hematopoietic cells including blood group antigens and complement mediator proteins. Deficiency of these proteins renders the RBCs vulnerable to excessive complement damage and hemolysis. In particular, loss of CD55 and CD59 complement regulators is recognized as the main cause leading to several manifestations of the disease that includes intravascular hemolysis and kidney damage [32,33]. Lack of CD55 is followed by increased opsonization of RBCs and subsequent phagocytosis and removal by reticuloendothelial cells in an extravascular manner. In contrast, the absence of CD59 leads to unrestrained formation of the membrane attack complex of complement system followed by a classic type II hypersensitivity damage to RBCs and intravascular hemolysis [33]. Importantly, kidney disease has been proposed to be a leading cause of mortality in this group of patients. Kidney disease associated with PNH was first described in 1971 [34]. A subsequent study of 21 patients with PNH revealed substantial incidence of functional and anatomic renal abnormalities encompassing hyposthenuria, tubular dysfunction, and declining glomerular filtration rate [35]. Importantly, pathological findings are very similar to sickle cell disease, which underscores the increased heme and hemosiderosis burden in the kidneys. This notion is also supported by presence of hemolysis in almost all patients that presented with AKI and PNH [36]. While these facts highlight the underlying unfettered heme as the main culprit of kidney disease in patients with PNH, it is obvious that the nonspecific and variable presentation and degree of hemolysis account for an overall lack of clinical reports of renal involvement in PNH [37]. As mentioned, clinical presentation of PNH is highly variable and dependent on multiple factors, and hence requires a high degree of suspicion for accurate diagnosis [38,39]. The presenting symptoms may include fatigue, chest pain, hemoglobinuria, dyspnea, abdominal pain, thrombosis and renal dysfunction, among others [39]. Once PNH is suspected, flow cytometry has emerged as the main diagnostic tool where patient’s peripheral blood cells are analyzed for reduction in or absence of CD55 and CD59, among other GPI-anchored proteins [40]. Treatment of PNH is based on the specific PNH category which itself is primed by the severity of hemolysis-associated symptoms and degree of bone marrow failure.

### 2.4. Malaria

**Figure 2:**
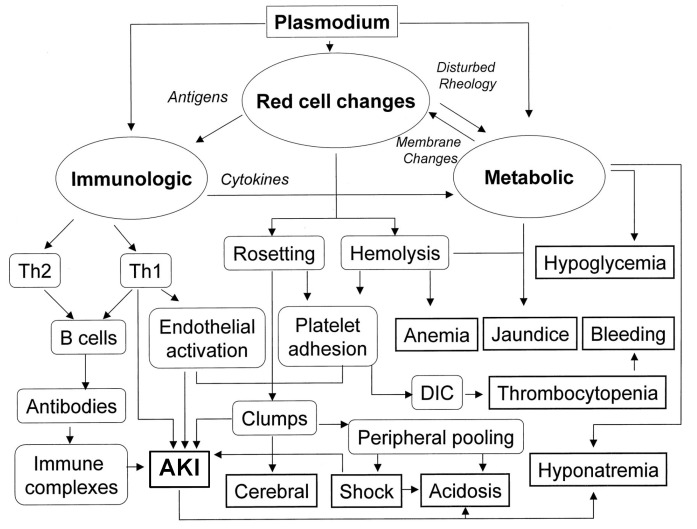
Pathways involved in pathogenesis of severe malaria. Modified from 44, with permission.

The bite of the infected female *Anopheles* mosquito is the instigating factor of an endemic disease with far reaching consequences. While malaria is endemic throughout most tropical areas, some rare transmission cases have also been documented that encompass rare congenitally acquired disease, blood transfusion, sharing of contaminated needles, and organ transplantation [41,42]. Following the bite, the parasites move to the liver and the infected individual may remain asymptomatic for up to 35 days until the erythrocytic stage of the parasite life cycle [43,44,45]. Once the infected red blood cells rupture and release the merozoites, the individual will experience fever along with other frequent manifestations of malaria, [43,44]. These may include generalized malaise, impaired consciousness, respiratory distress, pallor, renal and hepatic failure, coagulopathy, severe hypoglycemia and metabolic acidosis [43,44]. However, it is important to note that the clinical manifestations of malaria may vary depending on the parasite species, degree of immunity, and age. The most common pathogens that infect humans are *Plasmodium falciparum*, *Plasmodium ovale* and *Plasmodium vivax* [41]. The established diagnosis of malaria is based on consistent symptoms, and a positive malaria diagnostic test. The diagnostic tools comprise light microscopy of blood smears and rapid diagnostic tests [46,47]. Notably, if initial diagnostic assessment is negative while the clinical suspicion remains high, follow-up testing should be performed each day for two more days [47]. Pathogenesis of malaria is complex and involves various independent and overlapping pathways and is summarized in Figure 2. While severe malaria may involve different organs, renal involvement is a frequent complication and is recognized as an independent risk factor for death [48]. Within the kidney, different underlying mechanisms that culminate in kidney injury and disease have been proposed. Notably, malaria has been recognized to damage virtually all segments of the nephron, namely glomeruli, tubules and interstitium [48,49]. These findings have been corroborated in histopathological samples [50,51]. Classical indicators of renal involvement that include elevation of serum creatinine and blood urea nitrogen, proteinuria, microalbuminuria and urinary casts are often seen [49]. Although rare, cases of nephrotic syndrome have also been reported [52]. Overall, AKI, likely resulting from overt acute tubular necrosis, is the predominant finding when kidneys are affected [48,53,54]. While the precise mechanisms that culminate in the pathogenesis of malaria-induced kidney disease are not fully described, multiple pathways and mechanisms have been demonstrated to have fundamental significance in this context. These include intravascular hemolysis and release of heme moiety, endothelial activation, hemodynamic changes and microvascular dysfunction, parasite sequestration, exaggerated proinflammatory response, and reactive oxygen species (ROS)-mediated oxidative stress [43,48].

## 3. Molecular Insights

### 3.1. Heme: The Requisite for Aerobic Life

Heme is a complex of iron with protoporphyrin IX with fundamental biological functions for all aerobic cells. The inimitable function of heme is evidenced by its synthesis in all nucleated cells. By serving as the prosthetic group of many hemoproteins, it participates in diverse biological functions ranging from electron transportation, gas carriage, gas sensors and working as a cellular messenger [7,55,56]. Apart from hemoglobin and myoglobin, many other proteins also depend on heme to carry their vital functions that include cytochrome P450 enzymes, cytochromes involved in mitochondrial respiration, nitric oxide synthasesas, catalases, and peroxidases, histidine kinases, nitrophorins, among others [2,55]. Biosynthesis of heme is complex, involves multiple enzymes that work in concert inside the mitochondria and cytoplasm with the mitochondrial δ-aminolevulinic acid synthase serving as the rate-limiting enzyme [7]. The biosynthesis of heme is also heavily dependent on the rate of heme catabolism. Under physiological conditions amount of free heme is minimal and multiple evolutionary mechanisms have evolved to curtail availability of free heme following its release after injury. Given the vast amount of heme present in hemoglobin and myoglobin, it is not surprising that hemolysis and rhabdomyolysis are the two main culprits that lead to significant release of heme from damaged cells [57]. Notably, extracellular hemoglobin and myoglobin are easily oxidized leading to the transition of iron from a ferrous to a ferric state within the heme prosthetic group and subsequent release of heme culminating in significant rise of free heme [58,59,60]. Oxidation of heme moieties in hemoglobin to ferryl states also occur in vivo [61]. Ferric hemoglobin was demonstrated to lose heme moieties more readily than ferryl hemoglobin [62]. The extracellular hemoglobin and heme are readily scavenged by haptoglobin and hemopexin, respectively [63]. Ferryl hemoglobin is taken up via phagocytosis as well as CD163 receptor-mediated endocytosis [61]. The significance of scavenging free heme and the subsequent mitigation of heme’s injurious effects is well documented in a number of clinical settings, including, but not limited to, sickle cell disease and malaria [64,65,66,67]. These studies provide irrefutable evidence that not only support the injurious effects of free heme but also strategies that improve clinical outcomes by scavenging unfettered heme. However, this scavenging capacity can become exhausted, and subsequently free heme can accumulate in circulation. The hydrophobic nature of heme allows for its passage across cell membranes and subsequent buildup within the hydrophobic milieu of intact cells. A strong body of evidence demonstrates that free heme can cause direct and indirect injurious effects [4,24,57,60,68]. Such injurious effects are mediated by the pro-oxidant, pro-inflammatory and cytotoxic properties of heme. The pro-oxidant attributes of heme are multifaceted and include the generation of alkoxyl and peroxyl radicals that can further trigger lipid peroxidation [69,70]. Additionally, iron at the core of heme can participate in the Fenton reaction with subsequent formation of hyroxy-radicals [71]. Furthermore, heme also contributes to the formation of free radicals via enzymatic reactions of NADPH oxidases [4]. The peroxides generated during the mitochondrial respiration may also aggravate the pro-oxidant effects of heme [72]. The pro-inflammatory effects of heme are also well recognized [73,74,75,76]. While macrophages have been shown to secrete tumor necrosis factor in response to addition of heme in vitro [77], in vivo administration of heme results in increased vascular permeability, adhesion molecule expression, and leukocyte recruitment [76]. Furthermore, heme can also induce monocyte chemoattractant protein-1 [75] and enhance endothelial cell adhesion molecule expression [78], causing the activation of polymorphonuclear leukocytes and endothelial uptake of heme results in heightened damage by these activated cells [79,80]. The interplay between heme and macrophage-mediated inflammatory response and consequent kidney injury was elegantly described in a model of rhabdomyolysis in mice. This study demonstrated how macrophages contribute to the pathogenesis of rhabdomyolysis by releasing extracellular traps that are comprised of DNA fibers and granule proteins. Importantly, heme-activated platelets released from necrotic muscle cells during rhabdomyolysis augmented the production of these extracellular traps through increasing intracellular ROS formation as well as histone citrullination [81]. The relationship between heme and complement activation during rhabdomyolysis is also documented. Specifically, evidence supports a key role for myoglobin-derived heme activation of the alternative pathway arm of complement system [82]. It is evident that heme is a potent damage-associated molecular pattern. Exposure of neutrophils to heme is associated with multiple inflammatory characteristics. These include the organization of cytoskeleton in a manner that is consistent with phagocytic and migratory activities, increase in generation of neutrophil extracellular trap, generation of ROS via NADPH oxidase activity, upregulation of neutrophil survival factors such as IL-8, and stimulation of ERK, PI3K–Akt and nuclear factor-κB signaling pathways, all of which indisputably prolong inflammation [68,83,84]. Overall, while heme is a cardinal necessity for aerobic life, a strong body of evidence points towards its potential hazardous effects and obviates the demand for an overhaul mechanism to mitigate these effects, namely the HO-1/ferritin system.

### 3.2. Heme Oxygenase-1/Ferritin

Heme oxygenases (HO) are comprised of two isozymes, HO-1 and HO-2, that are responsible for catalyzing degradation of heme into ferrous iron, CO and biliverdin, the latter subsequently converted to bilirubin by the enzymatic reaction of biliverdin reductase [12]. While the expression of HO-1 is induced in response to a variety of endogenous and exogenous stimuli, HO-2 is a constitutive isoform of HO family and is expressed largely in the brain, kidney, and testis [12]. The byproducts of HO reaction were initially considered to be mere waste products. However, interest and robust investigations in the past three decades have affirmed that CO, biliverdin, bilirubin and iron induced ferritin expression are important mediators of many biological functions such as apoptosis, autophagy, immune cell trafficking, mitochondrial homeostatic integrity, regulation of innate and adaptive immunity, regulation of cell cycle, and angiogenesis, among others [5,6,12]. These observations were made possible thanks to a number of seminal breakthroughs. First, Nath and colleagues reported that the induction of HO-1 coupled to ferritin synthesis is a rapid, protective antioxidant response in a model of rhabdomyolysis induced AKI [10]. This was followed by observations reported by Balla and colleagues that induction of ferritin was protective in endothelial cells that were exposed to oxidant mediated injury [11]. Next, the generation of HO-1 knockout mice that evinced multiple pathological manifestations further substantiated the relevance of this enzyme system in the context of health and disease and delivered a platform for the investigations that followed [85]. The first description and diagnosis of a young patient with HO-1 deficiency that was reported by Yachie and colleagues revealed many similarities to the HO-1 knockout mice [86]. This rare, autosomal recessive disease has been since reported in a small number of other cases that underscores the indispensable nature of this enzyme [87]. The protective effects of HO-1 induction in diseases leading to increased heme burden are intricate. Importantly, under physiological conditions, the expression of HO-1 is suppressed via transcription factor, Bach-1 [88]. Induction of HO-1 expression not only leads to prompt degradation and removal of pro-oxidant and pro-inflammatory heme, but also generates molecules that are now well recognized to possess cytoprotective properties. Notably, there is also evidence that these cytoprotective effects may also be independent from the enzymatic activity of HO-1 and may also rely on nuclear localization and activation of transcription factors essential during oxidative stress [89]. As it relates to the immune system, it was demonstrated that macrophages lacking HO-1 expression are able to perform erythrophagocytosis, but this was followed by the rupture and death of macrophages leading to the release of oxidized heme and worsening inflammation [90]. The relevance of myeloid HO-1 expression is highlighted in other studies where it was shown that myeloid HO-1 controls the activation of interferon-regulatory factor-3 after Toll-like receptor 3 or 4 stimulation, or viral infection, suggesting that HO-1 plays a critical function in innate immunity [91]. The generated CO exerts beneficial effects in a number of injury models including hyperoxia, ischemia-reperfusion mediated injury, graft rejection, malaria, and sepsis among others [92,93]. Many cellular mechanisms have been proposed that mediated such favorable outcomes. CO is known to exert anti-inflammatory, anti-apoptotic and pro-phagocytic properties [93]. CO is shown to induce autophagy [94], inhibit mitochondrial dysfunction and inflammasome activation in macrophages [95], modulate mitochondrial biogenesis [96], and enhance acceleration of resolution of inflammation through biosynthesis of specialized pro-resolving mediators [97]. Biliverdin, another byproduct of the HO enzymatic reaction, is readily converted to bilirubin by the action of biliverdin reductase. Several salutary effects, anti-mutagenic, an antioxidant, anti-inflammatory, and immunosuppressant have been attributed to biliverdin [98]. Additionally, bilirubin has long been recognized for its potent anti-oxidant properties [99]. Overall, the cytoprotective properties of biliverdin and bilirubin have been established in a number of injury settings that include ischemia-reperfusion, graft rejection, sepsis, and intimal hyperplasia [100,101]. Ferritin also plays a paramount protective role in injurious settings. The ferritins are a family of proteins characterized by highly conserved three-dimensional structures similar to spherical shells, designed to sequester and store large amounts of iron in a safe, soluble and bioavailable form. It is made of 24 subunits of two types (FtH, heavy and FtL, light chain) whose proportion depends on the iron status of the cell, the tissue and the organ [8,102]. The indispensable functions of FtH are underscored by the embryonic lethality of mice with global deletion of FtH [103]. We and others have demonstrated the unique nephroprotective role of H-ferritin in different models of injury as well as its role in mitigating osteoblastic transition of cells that are evoked by uremic milieu [104,105,106,107]. Moreover, it must be noted that global HO-1 deletion, and targeted deletion of HO-1 and FtH in proximal tubules did not result in any significant abnormality in kidney function, as evidenced by normal serum creatinine levels as well as lack of proteinuria. Nevertheless, deletion of HO-1 and FtH is accompanied by substantial susceptibility to kidney disease in different forms of injury models [107,108,109,110].

## 4. Evidence to Support Salutary Effects of HO-1/Ferritin System against Heme-Protein Induced Kidney Disease

### 4.1. Rhabdomyolysis

The first line of defense against potential toxicity of unstable, oxidized hemoglobin and heme following hemolysis are haptoglobin and hemopexin, respectively [63]. However, as discussed earlier, this line of defense can be overwhelmed and saturated during massive hemolysis leading to increasing levels of free heme. It is of note that, to the best of our knowledge, no protein has been identified with the capacity to bind myoglobin following its release from disrupted muscle. Evidence that supports the crucial role of HO-1 has accumulated over the past three decades. Using hypertonic glycerol to induce rhabdomyolysis, Nath and colleagues were the first to demonstrate that inhibition of HO activity resulted in heightened injury, while prior conditioning of the animals with hemoglobin conferred protection [10]. These observations were later supported by similar experiments in animals with global deletion of HO-1 where deficiency of HO-1 was coupled with irreversible AKI and 100% mortality [111]. Importantly, an eight-fold increment in renal content of heme was observed in knockout animals, which further corroborates the adverse effects of free heme. In another study, using humanized HO-1 mice, where the human HO-1 gene, along with all its regulatory region, was inserted into mice with global HO-1 deficiency, it was revealed that the human HO-1 was functional, able to rescue the phenotype of HO-1 deficiency and conferred significant protection against rhabdomyolysis-induced AKI [112]. Wei and colleagues sought to investigate the mechanisms that govern the beneficial effects of granulocyte colony-stimulating factor (G-CSF) and found that such effects of G-CSF during glycerol induced AKI were dependent on HO-1. They found that G-CSF potently induced HO-1 in both cultured tubular cells and mouse kidneys, and that chemical inhibition of HO-1 was associated with mitigation of the protective effects of G-CSF [113]. Boddu et al. described a leucine-rich repeat kinase 2 (Lrrk2) deletion model of Parkinson’s disease in rats, wherein hemoglobin accumulation in kidney results in concomitant induction of HO-1. The Lrrk2 knockout rat model was protected against glycerol-induced rhabdomyolysis and preconditioning of these rats with endogenous hemoglobin conferred protection against AKI [114]. The protective effects of curcumin (active component in turmeric rhizomes and a known anti-oxidant), was also shown to at least in part confer protection during glycerol induced AKI via the upregulation of HO-1 [115]. The relevance of ferritin expression in the context of protective effects of HO-1 induction was highlighted in a study with targeted deletion of H-ferritin in proximal tubules (more details below) [107]. The non-redundant protective role of ferritin and hazardous role of free iron are supported by studies using deferoxamine (DFO), a potent iron cations chelator. Shah and colleagues demonstrated the negative effects of hydroxyl radical in glycerol-induced AKI and established that limiting iron availability via DFO was protective against AKI in this model [116]. Similarly, Paller’s studies showed that DFO administration was protective in two experimental models of pigment-induced AKI, intramuscular glycerol injection and intravenous hemoglobin infusion without and with concurrent ischemia in the rat [117].

### 4.2. Malaria

HO-1 induction and its corresponding advantageous effects were first shown to protect against experimental cerebral malaria [118]. In this study, genetic deletion of HO-1 and its enzymatic inhibition were associated with a higher incidence of cerebral malaria. Conversely, pharmacological induction of HO-1 and CO administration reduced such incidence and diminished blood–brain barrier disruption, brain microvasculature congestion and neuroinflammation. Mechanistic studies revealed that the binding capability of CO to cell-free Hb preventing heme release is a major underlying mechanism of these protective effects [118]. These findings and other investigations strongly indicate that free heme is a major culprit of severe malaria [67]. These protective effects have been validated in other studies and extend to mitigation of acute lung injury [119] as well as clinical outcome in pregnancy-associated malaria [120]. More recently, using various transgenic models, it was demonstrated that the development of disease tolerance to malaria is achieved via a damage control mechanism operating specifically in proximal tubular cells. Such tolerance was shown to be dependent on HO-1 and FtH expression, a mechanism that implicates the transcription-factor nuclear-factor E2-related factor-2 (NRF2). Overall, proximal tubular upregulation of HO-1 and H-ferritin detoxify free heme in proximal tubules thus alleviating development of a hallmark of severe malaria, namely AKI [121].

### 4.3. Sickle Cell Disease (SCD)

Vaso-occlusion and hemolysis are the clinical hallmarks of SCD and patients with SCD are at increased risk of developing chronic kidney disease [27]. As a consequence of frequent hemolysis, triggered by sickling (formation of rigid strands within the RBC that gives its characteristic, sickle shape) of affected RBCs, free hemoglobin and heme are released into circulation. During the initial phase, haptoglobin and hemopexin confer the first line of protection, but once saturated, the levels of free hemoglobin and heme significantly rise in the blood of SCD patients [122,123]. Nath and colleagues established that transgenic sickle mice display renal enlargement, increased heme content and HO activity, as well as medullary congestion. The human sickle kidney also shows similar findings with induction of HO-1 in renal tubules, interstitial cells, and in the vasculature [124]. Other investigators have validated these results by demonstrating HO-1 induction in different SCD mouse models [125,126]. Belcher and colleagues provided additional evidence to support the pivotal role of HO-1 in prevention of vaso-occlusion in transgenic sickle mice [127]. While there was a systematic induction of HO-1, treatment with hemin resulted in further overexpression of HO-1 accompanied by a reduction in hemostasis, leukocyte-endothelium interactions, and inhibition of some key pro-inflammatory proteins such as NF-κB, VCAM-1, and ICAM-1 expression [127]. Furthermore, while the pharmacological inhibition of HO activity led to the worsening stasis in these mice, protective effects of HO-1 induction were mimicked by HO byproducts biliverdin and CO. In fact, it has been postulated that increased levels of CO are the main driving force behind hyperperfusion and hyperfiltration which is frequently observed in SCD patients [128,129]. These effects are likely due to offsetting the vasoconstriction associated with nitric oxide depletion [130]. In humans, a variable GT-repeat length polymorphism in the HO-1 gene promoter is inversely correlated to HO-1 expression [131]. Saraf and colleagues demonstrated that in patients with SCD, homozygosity of the long allele (leading to lower levels of HO-1 expression) was associated with decreased glomerular filtration rate. In addition, the authors found enrichment of single nucleotide polymorphism downstream of HO-1 in sickle patients that was associated with chronic kidney disease stage or end stage kidney disease [123].

### 4.4. Other Models of Hemolytic Anemias and Protective Role of HO-1

As it conveys to inducing hemolysis in animals, Phenylhydrazine (PHZ) has been frequently utilized. PHZ is a potent chemical which causes significant toxicity in various tissues at different levels. However, it has also emerged as the most commonly used agent to induce intravascular hemolysis, as it results in significant membrane lipid peroxidation followed by massive hemolysis. Several studies have used this model and elaborated on the mechanisms of HO-1 mediated protection. These studies were recently summarized and discussed by Grunenwald and colleagues [132].

### 4.5. Ferritin and Nephroprotection

Increasing heme burden leads to the eventuality of increased iron release from heme moiety. Iron, the second most abundant element on earth, has the ability to change its valence and hence serve as a unique contributor in multiple diverse biological pathways. The ferrous iron released from heme degradation can readily participate in Fenton’s reaction, leading to the generation of ROS, which may induce the oxidation of proteins, lipids and lipoproteins, nucleic acids, carbohydrates and other cellular components [71]. The ability of free iron to exacerbate kidney injury is well documented as its chelation via deferoxamine is shown to be protective in injury models that are coupled with increased heme burden [116,117]. Moreover, Zager and colleagues delivered evidence using different models of kidney injury that upregulation of ferritin may serve as an advantageous “preconditioning” state [68,133]. There is also mounting clinical substantiation to suggest that higher plasma catalytic iron levels are associated with worse outcomes including AKI and need for kidney replacement therapy [134,135]. We have shown that targeted deletion of FtH in proximal tubules leads to heightened injury in glycerol-induced rhabdomyolysis, as supported by higher serum creatinine and mortality, as well as worse histological manifestations. Intriguingly, we also showed that deletion of FtH resulted in markedly higher levels of HO-1 expression, particularly post-injury [105]. Despite this increase in HO-1 expression, mice with deletion of proximal tubule-specific FtH succumbed significantly more frequently following rhabdomyolysis. This observation unequivocally demonstrates that the salutatory effects of HO-1 expression are co-dependent on FtH. These findings are corroborated in another study where FtH overexpression, through doxycycline-inducible system, led to lesser apoptosis and improved tubular viability, likely through mitigating the oxidative stress [136]. Moreover, it has been shown that systemic and locally generated hepcidin protect against hemoglobin-induced AKI. Through subsequent studies, Scindia and colleagues recapitulated the protective effects of hepcidin in AKI and postulated that hepcidin exerts such effects through increased iron retention with subsequent upregulation of intracellular ferritin [137]. Similar results have been recapitulated in a model of hemoglobin-mediated nephropathy [138]. As mentioned above, recent studies underscored the significance of proximal tubular HO-1 and FtH induction in a malaria model. Using transgenic mice with targeted deletion of FtH or HO-1, it was shown that these proteins are critical to establish disease tolerance to malaria by detoxifying the free heme and diminishing development of AKI [121]. 

## 5. Conclusions and Future Directions

While both heme and iron as its core participant are essential for living organisms, their unfettered labile forms may have detrimental consequence at cellular and organ level that also involves the kidneys. Despite significant progress in the field of HO-1 and ferritin since the discovery of their protective effects in early 1990s, a full transition from bench to bedside remains elusive. This is due to several factors that include potential the side effects of byproducts, difficulty conveying the induction of HO-1 and ferritin at the desired organ/site, and major differences between the molecular regulation of the mouse and human HO-1 genes. Nevertheless, with increasing advancement and availability of transcriptomic and proteomic analysis tools, high-throughput screening, and generation of humanized HO-1 mice that harbor the entire human HO-1 gene along with all its regulatory regions, we may be converging on major breakthroughs that would allow for timely and precision-based therapies by targeting HO-1 and ferritin.

## Figures and Tables

**Table 1:** Partial list of different insults/mechanisms that lead to hemolysis.

Causes of Hemolytic Anemias
**Intrinsic (intracorpuscular)** A. Genetic defects − Disorders affecting the cytoskeleton of red blood cell membrane (e.g., spherocytosis) − Glucose-6-phosphate dehydrogenase deficiency − Glutathione synthetase deficiency − Pyruvate kinase, hexokinase deficiency − Deficient globin synthesis: Thalassemia syndromes − Abnormal globin structure: Sickle cell anemia B. Membrane defects: Paroxysmal nocturnal hemoglobinuria **Extrinsic (extracorpuscular)** A. Antibody mediated − Isohemagglutinins: Transfusion reactions, erythroblastosis fetalis − Autoantibodies: idiopathic, lupus, drug associated (e.g., quinidine, α-methyldopa) B. Traumatic/mechanical damage − Thrombotic thrombocytopenic purpura − Disseminated intravascular coagulation − Extracorporeal circulation − Prosthetic valves C. Infections: malaria, clostridia D. Chemical agents: Arsine, Glycerol, Benzene, Sodium chlorate, Methyl chloride, etc. E. Venoms: Rattle snake, Coral snake, Brown recluse spider

## Data Availability

Not applicable.
